# Decreasing the Surgical Errors by Neurostimulation of Primary Motor Cortex and the Associated Brain Activation via Neuroimaging

**DOI:** 10.3389/fnins.2021.651192

**Published:** 2021-03-22

**Authors:** Yuanyuan Gao, Lora Cavuoto, Anirban Dutta, Uwe Kruger, Pingkun Yan, Arun Nemani, Jack E. Norfleet, Basiel A. Makled, Jessica Silvestri, Steven Schwaitzberg, Xavier Intes, Suvranu De

**Affiliations:** ^1^Center for Modeling, Simulation and Imaging in Medicine, Rensselaer Polytechnic Institute, Troy, NY, United States; ^2^Jacobs School of Medicine and Biomedical Sciences, University at Buffalo, The State University of New York, Buffalo, NY, United States; ^3^Department of Biomedical Engineering, University at Buffalo, The State University of New York, Buffalo, NY, United States; ^4^Department of Biomedical Engineering, Rensselaer Polytechnic Institute, Troy, NY, United States; ^5^U.S. Army Combat Capabilities Development Command – Soldier Center (CCDC SC), Orlando, FL, United States; ^6^SFC Paul Ray Smith Simulation and Training Technology Center (STTC), Orlando, FL, United States; ^7^Medical Simulation Research Branch, Orlando, FL, United States; ^8^Department of Surgery, University at Buffalo, The State University of New York, Buffalo, NY, United States; ^9^Buffalo General Hospital, Buffalo, NY, United States

**Keywords:** motor learning, neurostimulation, neuroimaging, functional near-infrared spectroscopy, surgical performance

## Abstract

Acquisition of fine motor skills is a time-consuming process as it is based on learning via frequent repetitions. Transcranial electrical stimulation (tES) is a promising means of enhancing simple motor skill development via neuromodulatory mechanisms. Here, we report that non-invasive neurostimulation facilitates the learning of complex fine bimanual motor skills associated with a surgical task. During the training of 12 medical students on the Fundamentals of Laparoscopic Surgery (FLS) pattern cutting task over a period of 12 days, we observed that transcranial direct current stimulation (tDCS) decreased error level and the variability in performance, compared to the Sham group. Furthermore, by concurrently monitoring the cortical activations of the subjects via functional near-infrared spectroscopy (fNIRS), our study showed that the cortical activation patterns were significantly different between the tDCS and Sham group, with the activation of primary motor cortex (M1) and prefrontal cortex (PFC) contralateral to the anodal electrode significantly decreased while supplemental motor area (SMA) increased by tDCS. The lowered performance errors were retained after 1-month post-training. This work supports the use of tDCS to enhance performance accuracy in fine bimanual motor tasks.

## Introduction

From learning to play the violin to performing delicate surgery, perfecting fine motor skills requires significant repetitive practice (Adams, 1987). The training may take days, months, and even years. Sometimes, despite repeated practice, the resulting skill level might remain low (Grantcharov and Funch-Jensen, 2009; Gao et al., 2020b). Deliberate practice, i.e., purposeful practice that requires focused attention and feedback, has been proposed as a learner-centric approach to accelerate performance (Bell and Kozlowski, 2010). However, the use of novel technology to enhance fine motor skills remains limited.

Recently, neuromodulation has been proposed to enhance motor skill learning. This is motivated by the finding that motor learning involves neuroplasticity (Karni et al., 1995). It is also shown that motor learning recruits multiple brain areas (Floyer-Lea and Matthews, 2005). Transcranial electrical stimulation (tES) is a neuromodulation technique that can affect neuroplasticity and can facilitate motor learning. It changes the excitability of the cortex by delivering a small amount of current using electrodes attached to the scalp. Studies have shown that tES improves human motor learning, including visuospatial learning, sequence learning, and adaptation, in its direct current form, transcranial direct current stimulation (tDCS). For example, during a 5-day training program, the primary motor cortex (M1) region tDCS increased the performance scores for a visuospatial task (Reis et al., 2009). In another 3-day training program, M1 tDCS increased performance scores for both sequence and visuospatial learning (Saucedo Marquez et al., 2013). More recently, transcranial random noise stimulation (tRNS) has shown to improve learning (Terney et al., 2008). The study confirmed that applying tRNS benefited the reaction time for a finger-tapping task (Terney et al., 2008). However, further studies are scarce.

Most tES studies have focused on simple unimanual motor sequence learning. The effect of tES on complex motor skills, such as bimanual motor skills, remains relatively under-studied. Although complex motor skills may be decomposed into simpler motor tasks (Willingham, 1998), higher-level coordination is involved (Swinnen and Wenderoth, 2004). Furthermore, the learning procedure for complex skills is usually time and resource-consuming. Here, to the best of our knowledge, we are the first to investigate the effect of tES on the learning procedure of a complex surgical motor task (Patel et al., 2020), which typically takes more than 10 days to achieve proficiency (Gao et al., 2020c).

Alongside the performance change in the motor learning procedure, we assessed the brain activation change considering its association with motor learning neuroplasticity. Prior fMRI studies (Floyer-Lea and Matthews, 2005) show that specific cortical areas are activated during the motor learning stages, including the prefrontal cortex (PFC), supplementary motor area (SMA), and M1 regions. To acquire the brain activation changes, we used a non-invasive functional brain imaging technique, functional near-infrared spectroscopy (fNIRS). This fNIRS technique has been widely used in other motor skill studies (Nemani et al., 2018). In addition to high temporal- and spatial-resolution, fNIRS can be coupled with tES during motor tasks without constraining or interfering with motor task execution (McKendrick et al., 2015).

In our study, we tested the hypothesis that tDCS of the primary motor cortex will facilitate complex surgical motor skill learning. We report the behavioral metrics in initial learning, consolidation learning, and skill retention. The second hypothesis is that the tDCS changes brain activation. By testing the two hypotheses, we report the effects of M1 tDCS to be reducing the performance error, as well as stabilizing the trial-to-trial variability, in conjunction with brain activation changes in the motor learning related cortex regions.

## Materials and Methods

### Participant Recruitment

This study was approved by the Institutional Review Boards of the University at Buffalo and Rensselaer Polytechnic Institute. All the participants provided written informed consent to take part in the study. All the participants were novices to the bimanual task, as they had no experience with laparoscopic tools, Fundamentals of Laparoscopic Surgery (FLS) training, or any similar surgical simulation training software. We recruited 14 medical students. Among the 14 medical students recruited in this study, two subjects did not pass the CUSUM exam and were excluded from the data analysis (explained in the Supplementary Material “CUSUM scores”). The subjects were randomly divided into two groups at the beginning of the study: tDCS and Sham (demographics in Table 1). Since the standard deviation of age in tDCS group seemed greater than the other two groups, we further performed the Mann–Whitney *U*-test to compare the age value distribution of the two groups and found no significant difference (*p* = 1.000). The visualization of the age distribution is shown in Supplementary Figure 2. The ages of all the participants are between 20 and 30 years old, which satisfied the recruitment inclusion criterion of “above 18 and under 65 years old.”

**TABLE 1:** Participant demographics.

	**tDCS**	**Sham**
# of participants	5	7
Age (mean ± SD)	24.60 ± 3.36	24.00 ± 0.82
Sex (F:M)	4:1	5:2
Handedness (R:L)	5:0	7:0

### Power Analysis

From our previous study, 12 days of FLS pattern cutting task training showed clear learning curves for both FLS score and fNIRS metrics (Nemani et al., 2018). There was no previous study data that could support the power analysis to estimate the number of participants needed. Based on our previous study, an effect size was selected as Cohen’s f of 1.26 (Nemani et al., 2018). For the repeated measures analysis of variance (ANOVA) power analysis with a 95% confidence interval and a minimum power of 0.90, it was determined that a minimum of 8 as the total sample size (4 per group), calculated using the statistical software package G^∗^Power.

### Experimental Design

**FIGURE 1:**
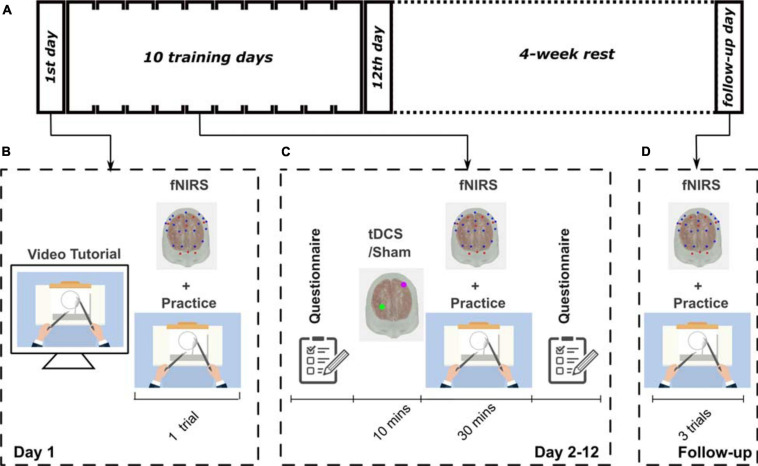
The experimental protocol designs. **(A)** Schematic showing the experimental design for this study. All participants went through a 12-day training procedure followed by a 4-week rest, and then attended a follow-up visit. **(B)** Illustration of the training of the first day; **(C)** from day 2 to day 12; and **(D)** on the follow-up visit.

The participants underwent 12 visits on 12 consecutive days and one visit as a follow-up visit 4 weeks later (Figure 1). On the first day, demographic information, including age and handedness, were collected. Participants were made familiar with the experimental apparatus, including the cap, optodes, and electrodes. The impedance of tDCS and the signal quality of fNIRS were checked to ensure they were within an acceptable range. All participants were instructed on how to perform the task through a standardized video tutorial^1^ and verbal instructions. The bimanual task was the pattern cutting task selected from the FLS program^2^ (Nemani et al., 2018). After the training, participants performed a single trial of the pattern cutting task to show that they fully understood the task and as a measure of baseline performance. From day 2 to day 12, the subjects underwent 10 min of the stimulation (tDCS or Sham according to their group assignment) and then practiced 30 min of the task, sequentially. While the participants were practicing the task, fNIRS imaged their brain activation. Four weeks after the completion of the training, subjects returned for a follow-up visit, during which they performed the same FLS pattern cutting task three times, to measure their skill retention. Participants completed a safety questionnaire (Thair et al., 2017) before and after each neuromodulation session. A photo of a participant during the experiment is shown in Supplementary Figure 3b.

### Motor Task and Task Performance Metrics

The participants practiced a bimanual motor task, pattern cutting, selected from the FLS program which is a pre-requisite for board certification in general and obstetrics and gynecology surgery. The bimanual motor task was performed using an FLS simulator (Laprascopic skills trainer, Limbs & Things, United Kingdom). Standard FLS-certified laparoscopic tools were used to cut a marked piece of gauze as quickly and as accurately as possible. The participants were instructed to avoid unnecessary movements of their body or facial muscles, and to refrain from speaking, to prevent motion artifacts in the fNIRS signals. The cap holding the fibers on the participant, as well as the wires, did not hinder the participant’s movement during the task.

The three performance quantification metrics were time, errors, and the FLS score. Time was captured starting from the moment when the tools touched the gauze and ending at the moment of completion. The error was counted as the area between the marked circle on the gauge and the actual cut. The FLS scores were determined using the standardized FLS scoring metric formulation for the pattern cutting task based on the time and error. This formulation is intellectual property–protected and was obtained with a nondisclosure agreement with the FLS Committee, and hence, its details cannot be reported in this paper.

### Transcranial Electrical Stimulation Hardware and Settings

The tES stimulation was delivered by a commercial device (StarStim, Neuroelectronics, Spain). Anodal electrode was placed over the C3 location, according to the international 10–20 system, aiming to simulate the left primary motor cortex (M1) region. The return electrode was placed at Pf2 over the right PFC region. The electrode was based on a sintered Ag/AgCL pellet with a 12 mm diameter, with a total electrode area of 1 cm^2^. Gel (Parker Laboratories, Inc.) was applied on the electrode to decrease the impedance and improve the signal quality. A trained researcher carefully adjusted the contact between the electrode and the scalp until the impedance was lower than 15 kΩ. During the stimulation protocol, the impedance was monitored every second. At any time, if the impedance exceeded 20 kΩ in any stimulation electrode, the stimulation protocol was aborted to protect the subject from the high voltages generated as an embedded function in StarStim. The stimulation lasted 10 min. tDCS was delivered at 1 mA. The current was ramped up for 30 s to 1 mA and down to zero current at the beginning and the end of the stimulation. Sham stimulation was set at zero current with the same ramp parameters as tDCS, imitating the same sensation to blind the subjects to the stimulation type (see Supplementary Figure 3d).

### NIRS Hardware and Equipment

We used a commercially available, continuous-wave near-infrared spectrometer, which delivered infrared light at 760 and 850 nm (NIRScout, NIRx, Berlin, Germany). The system used 8 LED light sources coupled to 19 long-distance detectors and 8 short-distance detectors. The combinations between the sources and the detectors resulted in 28 measurement channels. A schematic of the geometric arrangement of probes is in Supplementary Figure 3a. The probe design was derived from our previous study (Nemani et al., 2018), which was determined using Monte Carlo simulations and was characterized to have high sensitivity to functional changes in the PFC, M1, and SMA. The optode positioning and NIRS signal processing is in Supplementary Material.

### Statistical Methods

The mean of performance error, performance time, and the FLS score were compared between the two groups (tDCS and Sham) on each training day. The normality of the data was first checked by Kolmogorov–Smirnov test. If it passed the Kolmogorov–Smirnov test, the two-sided *t*-test was performed, otherwise the Mann–Whitney *U*-test was performed. A repeated measures ANOVA model was adopted when analyzing the effect of stimulation type and the training time points (pre-test, post-test, and retention) on the performance outcomes. For the repeated measures ANOVA model, Levene’s test was carried out to check for homoscedasticity, and Mauchly’s test was carried out to check sphericity. We tested each brain region separately by comparing the mean brain activation between groups. Either *t*-test or Mann–Whitney *U*-test was adopted based on the Kolmogorov–Smirnov test results. The correlation between performance and brain activation was analyzed using Pearson’s correlation coefficient. An alpha level of 0.05 was set as the minimum required to reject the null hypothesis, and further corrected by Bonferroni correction if multiple comparisons were carried out. All the *p*-values presented are original without correction. Descriptive and inferential statistics were performed in MATLAB and SPSS. All error bar plots display mean values along with a 95% confidence interval of the mean values.

## Results

### Behavioral Change in Motor Learning Due to Neuromodulation

**FIGURE 2:**
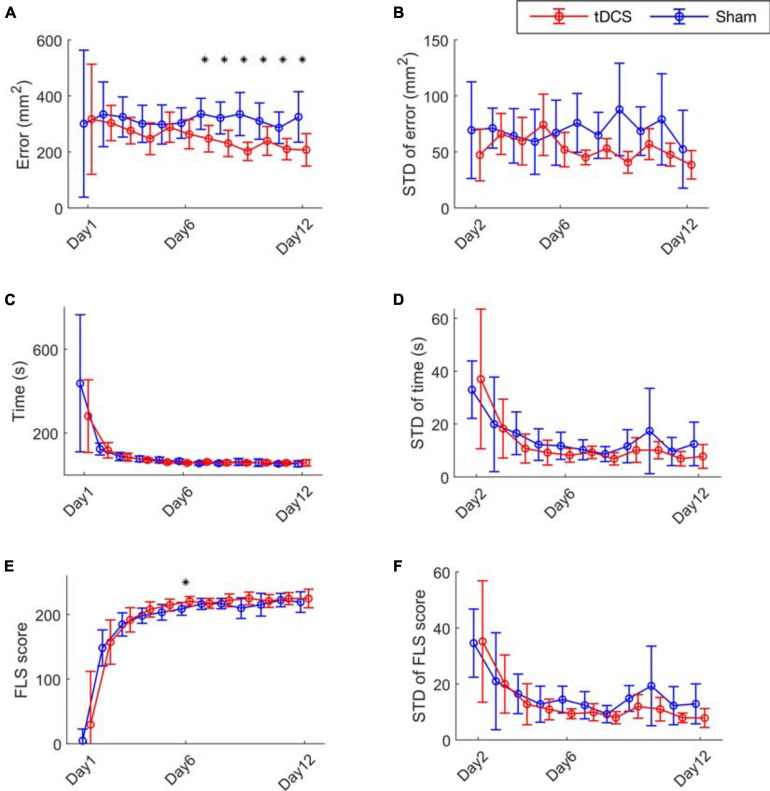
Bimanual motor task performance. **(A)** The cutting error defined as the cutting area deviated from the pre-marked circle on the gauze, **(C)** the performance time and **(E)** the FLS score for the 12 training days are presented. **(B)** The mean trial-to-trial standard deviation (STD) value of error **(D)** time, and **(F)** score from day 2 to day 12. The stars represent a significant difference compared to the Sham group (details are in Supplementary Table 1).

We first present the results analyzing the performance matrix (Figure 2 and Supplementary Table 1). The performance error of the tDCS group was significantly lower than the Sham group from day 7 to day 12 (Figure 2A). The mean trial-to-trial variability is quantified by the mean of the standard deviation of the performance error across subjects on each day (Figure 2B). Compared to the Sham group, the standard deviation values showed lowering trend in the tDCS group (not significant). We present the time and FLS score metrics in Figures 2C–F. The two groups had similar learning curves in time and FLS scores, with no significant difference found, except scores on day 6 (Figures 2C,E). The standard deviations of the performance time, and FLS score, were also calculated on day 2–12 (Figures 2D,F). The tDCS group performed lowering trend of trial-to-trial standard deviation of the performance time and FLS score than the Sham group without significance (not significant). The safety questionnaire results are in Supplementary Figure 4.

### Brain Activation Change During Training

**FIGURE 3:**
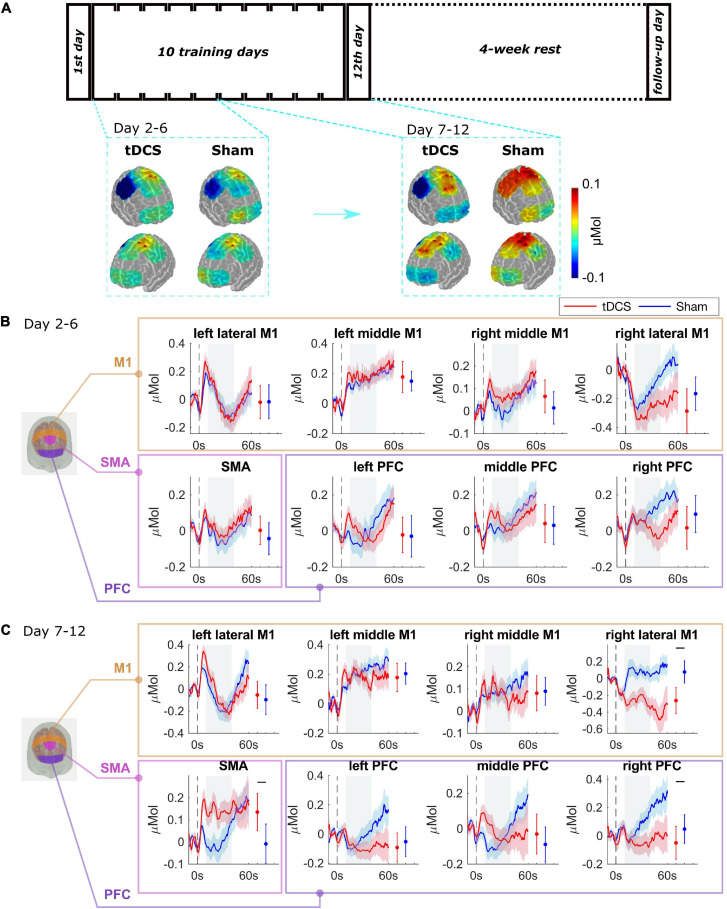
Brain functional activation during the training procedure. **(A)** The average of HbO changes is shown as spatial maps for PFC, M1, and SMA regions for training day 2–6, and day 7–12. **(B)** Grouped average time-series HRFs with respect to cortical regions on training day 2–6 and **(C)** day 7–12. The solid lines are mean values, and the shaded areas are 95% confidence interval. The stimulus onset begins at 0 s (dashed black line) indicating that the trial has started. Negative time indicates the baseline measurement used for calibration before each trial. The gray painted box (10–40s) is the time range selected to calculate the mean HbO values. The mean and 95% CI of 10–40s HRFs are plotted next to the time-series HRFs in error bar form. The black bar indicates significant difference (see Supplementary Table 2).

The average of oxy-hemoglobin (HbO) changes is shown as spatial maps during the training day 2–6, and during day 7–12 in Figure 3A. We did block average of brain activation for day 2–6 and day 7–12 because the performance error differed significantly between groups from day 7. The block averaging method is commonly adopted by the fNIRS community (Huppert et al., 2009; Pfeifer et al., 2018). Baseline activations on day 1 were not significantly different between two groups, see Supplementary Figure 5. For the Sham group, compared to the initial learning stage (day 2–6), and M1 region was activated to a higher degree during the later learning stage (day 7–12). The tDCS group showed a similar brain activation pattern as the Sham group in the learning stage, as the brain activation levels were not significantly different between the two groups during the initial learning stage (day 2–6) (see Figure 3B). For day 7–12 (Figure 3C), compared to the Sham group, the tDCS group showed higher activation level in SMA and lower activation level in right PFC and right lateral M1 region (right lateral M1 *p* = 0.001, SMA *p* = 0.001, right PFC *p* = 0.001). The most prominently depressed activation area, i.e., the right M1 region, was the cortical area contralateral to the tDCS anode. The full significance test results are shown in Supplementary Table 2.

### Skill Retention

**FIGURE 4:**
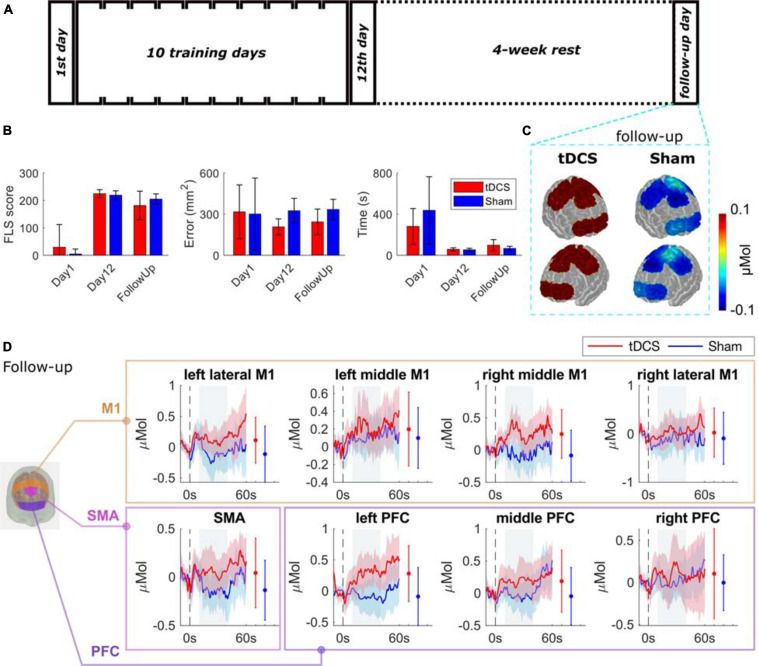
Bimanual motor task performance and functional brain activation for the follow-up tasks. **(A)** Experimental protocol. **(B)** The performance of the two groups on day 1, day 12, and the follow-up day visit in performance error, performance time, and the FLS score (details are in Supplementary Table 3). **(C)** The average HbO changes are shown as spatial maps for PFC, M1, and SMA regions for follow-up tasks. **(D)** Grouped average time-series HRFs with respect to cortical regions on follow-up visit. The solid lines are mean values, and the shaded areas are 95% confidence interval. The stimulus onset begins at 0 s (dashed black line) indicating that the trial has started. Negative time indicates the baseline measurement used for calibration before each trial. The gray painted box (10–40s) is the time range selected to calculated the mean HbO values. The mean and 95% CI of 10–40s HRFs are plotted next to the time-series HRFs in error bar form. The significance test results are in Supplementary Table 2.

The baseline performance (day 1), the post-test (day 12), and the retention (follow up performance) were analyzed, and the results are shown in Supplementary Table 3 and illustrated in Figure 4B. For the performance error, the repeated measures ANOVA showed that there was no significant difference between stimulation types (tDCS, and Sham; *F* = 2.830; *p* = 0.123), the effect of time points (day 1, day 12, and follow up visit; *F* = 0.944; *p* = 0.432), or the interaction of stim × time (*F* = 1.199; *p* = 0.327). For the performance time, only the effect of time point (*F* = 27.945; *p* = 0.001) was significant, but not the effect of stimulation (*F* = 0.071; *p* = 0.796), or the stim × time interaction (*F* = 2.534; *p* = 0.136). For the FLS score, there was a significant difference between the interaction of stim × time (*F* = 1.799; *p* = 0.048) and the time points (*F* = 114.367; *p* = 0.001), but not the stimulation (*F* = 0.024; *p* = 0.879).

The comparisons between the two groups of their brain activation map on the retention task are shown in Figure 4C with time-series results and significance test results in Figure 4D. We observed a trend of increasing brain activation in the tDCS group and a decrease in the Sham group, but the difference was not significant. The full significance test results can be found in Supplementary Table 2.

### Correlation Between Performance and Brain Activation

The correlation between performance and brain activation was further analyzed by Pearson’s linear correlation coefficient analysis. The results are shown in Supplementary Figure 6. The performance error showed significant negative correlation to middle PFC and SMA region activation. The left PFC, middle PFC, and left lateral M1 region brain activation and the performance time were also significantly correlated.

## Discussion

In this study, we examined the effect of the M1 tDCS on bimanual motor skill learning and retention. We also employed the fNIRS technique to acquire brain activation changes during task performance. We observed that tDCS lowered the performance error as the training proceeded. The trial-to-trial standard deviation analysis showed that the tDCS group tended to stabilize performance variability in performance error, time, and FLS score. The effect of tDCS in enhancing performance accuracy was retained after a 4-week break period. Accordingly, contralateral M1 and PFC brain activation were decreased and SMA was increased by tDCS. The correlation between performance error and middle PFC and SMA region activation was negative.

The effect that tDCS reduces performance errors observed in this study is significant since performance error is critical in surgical motor tasks. For example, surgery-related injuries were reported in 2.5 out of 1000 cases (Flum et al., 2001). The leading cause of maritime accidents was also reported to be human errors (49–85%) (Hetherington et al., 2006). From the learning curves shown in Figure 2, the learners decreased their performance time prominently, but error improvement occurred more slowly. In other words, the ceiling effect happened to time and score in an early stage, which was probably the reason why they were not affected by tDCS. Conversely, the error did not show ceiling effects in 12 days, and the tDCS had effects of decreasing the errors. Once trained, the lowered error level was retained after 4 weeks (Figure 4). These results indicate the merit of the application of tDCS to increase performance accuracy in fine motor skill learning. The benefit, risks, and ethical dilemmas have been discussed in a recent review paper (Patel et al., 2020).

Without any stimulation, in the Sham group, brain activation shifted to the bilateral M1 region with increased repetitions. Notably, fMRI studies have shown that the transition of short-term to long-term motor learning is featured by a shift of brain activation from anterior to more posterior brain regions (Floyer-Lea and Matthews, 2005; Nemani et al., 2018). Training to perform an explicit sequence of finger movements over several weeks showed progressively increasing blood-oxygen-level-dependent (BOLD) activity in M1 (Kami et al., 1995; Karni et al., 1998; Hluštík et al., 2004; Floyer-Lea and Matthews, 2005; Lehéricy et al., 2005; Xiong et al., 2009; Ma et al., 2010), interpreted as reflecting recruitment of additional M1 units into the local network that represents the acquired sequence of movements. In our previous study (Nemani et al., 2018), we showed that skilled learners had more brain activation in M1 compared to unskilled learners, as well as expert surgeons compared to novices. With tDCS, we found lower M1 activation contralateral to the anodal electrode, a phenomenon that has been reported in studies (Rothwell et al., 1992; Daskalakis et al., 2002; Tazoe et al., 2014). Other than the M1 region, we also observed increased activation in PFC and SMA region, which has also been documented in literatures that the tDCS could also affect the remote regions (Baudewig et al., 2001; Jang et al., 2009; Stagg et al., 2009, 2012). The neurophysiology mechanism of tDCS has been discussed extensively in the literature (see a review paper (Reed and Kadosh, 2018)). To summarize it, the long-term effects of tDCS depend on neurotransmitters that bind to receptors, such as glutamatergic (NMDA) and gamma-aminobutyric acid (GABA)-ergic receptors, as blocking these receptors resulted in suppression of the post-stimulation effects. In addition, glial cells and non-synaptic mechanisms may play a role in the afterward effects.

This study showed a successful combination of neuroimaging and neuromodulation. The multimodality of the two offered us valuable information that could not be derived with only one of them. The use of both neuroimaging and neuromodulation is an excellent way to investigate and understand neuromodulation (Gao et al., 2020a). However, here, we only report the observation of the cortical activation changes via neuroimaging. Closer and more in-depth combination of the two could help boost the effect of the neuromodulation, such as a neurofeedback loop and personalized tES (Gao et al., 2020a). Through these applications, the parameters of the neuromodulation could be regularized by the information from the neuroimaging technique. Motor performance could also be further optimized. This work contributes to this approach by offering the observation of the neural changes detected by neuroimaging under different neuromodulation conditions.

Another future direction is coupling neuromodulation with other techniques, such as EEG. fNIRS detects neurovascular changes in the cerebral cortex to reflect the brain activation level. However, brain activity includes other features, such as oscillatory, neuro-electrical, and neuro-chemical activities. The investigation in those areas could not be derived from fNIRS alone. Thus, introducing other measurements in the future could help understand the neuroscience mechanism of the neuromodulation, or the motor learning itself. Techniques other than neuroimaging are also beneficial. For example, the video data of the task execution or kinematic data of the tools also offer insights into motor learning.

In summary, we demonstrate that tDCS facilitates surgical bimanual motor skill learning by resulting in lower performance error. We further show decreased contralateral excitation in M1 and PFC contralateral to the anodal position and increased SMA excitation with tDCS compared to Sham. While existing metrics of task performance reward increased execution speed, our work sheds light on the importance of reducing errors, and the positive role of tDCS in achieving that outcome.

## Data Availability Statement

The data related to this study is available upon request to the corresponding author by signing a data transfer agreement. The code related to this study has been uploaded in https://github.com/YuanyuanGao216/NeuromodulationStudy.

## Ethics Statement

The studies involving human participants were reviewed and approved by the University at Buffalo and Rensselaer Polytechnic Institute. The patients/participants provided their written informed consent to participate in this study.

## Author Contributions

YG, AN, and SD designed the research. YG, LC, and JS performed the research. YG, UK, PY, and XI analyzed the data. YG wrote the manuscript. All authors contributed to the article and approved the submitted version.

## Conflict of Interest

The authors declare that the research was conducted in the absence of any commercial or financial relationships that could be construed as a potential conflict of interest.

## Footnotes

1 https://youtu.be/mUBZoSO3KA8

2 www.flsprogram.org
