# Supplementary material for: Decreasing the Surgical Errors by Neurostimulation of Primary Motor Cortex and the Associated Brain Activation via Neuroimaging
Source: Front Neurosci. 2021 Mar 22;15:651192. doi: 10.3389/fnins.2021.651192 (PMC8019915; doi:10.3389/fnins.2021.651192)
Supplement: Supplementary file 1 [file Data_Sheet_1.PDF]

Supplementary Material

1 Supplementary Methods

Participant recruitment method

Potential participants were recruited from the University at Buffalo's current medical student population through campus emails, social media, flyers, and word of mouth communication. We pre-screened 30 respondents through a pre-screening questionnaire (Thair et al., 2017). Four of them were excluded due to safety concerns or hairstyles that might prevent electrode or optode contact with the scalp. Two dropped out before consenting to participate, and three dropped out during the study due to personal reasons, leaving twenty-one medical students who completed the training protocol.

CUSUM scores

After the training, we plotted a CUSUM learning curve for each participant. In CUSUM analysis, positive or negative increments are added to a cumulative score according to the failure or success of the successive trial (Kestin, 1995). The pass/fail threshold was set at the average score they received on the first five trials (FLS score of 33), a method derived from Linsk et al.'s study (Linsk et al., 2018). This value is lower than 44 in the non-competent group in Fraser et al.'s. study (Fraser et al., 2003) and 39 in the novice group in the Chellali et al.'s study (Chellali et al., 2015). However, considering that in this study, we only recruited medical students without any experience, less experienced than the cohort of medical students and junior surgical residents in Fraser et al.'s study (Fraser et al., 2003) and surgeons of PGY1-4 in Chellali et al.'s study (Chellali et al., 2015), a lower threshold is reasonable. To calculate the CUSUM score, if a trial was a "pass," the respective CUSUM score is subtracted by 0.07; the CUSUM score was incremented by 0.93 in case of a "fail". Four subjects did not pass the desired acceptable failure rate of 0.05 and were excluded from the data analysis. A graphical representation of the CUSUM curve for each participant is in Fig. S1b. The brain activation pattern of the excluded subjects was in Fig. S1c. The pattern is similar to the follow-up task in Fig. 4c, in the sense that the brain cortex was over-activated and the activation was spread out through the cortex regions.

Optode positioning

A schematic of the geometric arrangement of probes is in Fig. S3a. The long-distance channels captured all measurements within a 30- to 40-mm distance between the source and the detector. The short-distance channels captured measures within an ~8-mm distance between the source and the detector. The short-distance channels were limited to probing the superficial tissue layers, such as skin, bone, dura, and pial surfaces, whereas the long-distance channels penetrated both superficial layers and cortical surface. We further added one channel to each of the SMA, the left, and the right lateral M1 regions to increase data measurement in those regions of particular interests. The optical probes were positioned carefully on the participant to avoid hair between the source/detector and scalp.

The method to ensure the optode position placement in this study was two-fold. First, we used a standard electroencephalography cap (EASYCAP: www.easycap.de) to hold the optodes (Nemani et

al., 2018). The cap had marked anatomical landmarks for placement on the scalp. The cap was carefully placed on the scalp by aligning the landmarks on the head. Second, we used a magnetic tracking device (trakSTAR, Ascension Tech Corp. Canada) to measure the positions of the optodes in 3D space. After we put the cap with optodes and electrodes on a participant, a 6 DOF sensor (diameter = 2mm) was placed on the positions of the optodes and electrodes to measure the 3D positions. The measured data were analyzed offline using the software package AtlasViewer to calculate the inter-trial standard deviation of the position for each optode to ensure that the optode was in the intended location. The mean positions are shown in Fig. S7 and Table S4.

NIRS signal processing

Data processing was completed using the open-source software HomER2 (Huppert et al., 2009), which is implemented in MATLAB (MathWorks). First, channels with light intensity greater than $10\ \mu V$, which indicates environmental light contaminated the signals, or smaller than $0.01\ \mu V$, which indicates the light was blocked by hair, were excluded. The channels with signal-to-noise ratio of light intensity greater than 3 were excluded as well due to poor signal quality. The remaining raw optical signals (intensity at 760 and 850 nm) were converted into optical density. Motion artifacts and systemic physiological interference were corrected using a third-order Butterworth low-pass filter with a cutoff frequency of 0.5Hz. The filtered optical density data were used to derive the concentration changes of oxyhemoglobin and deoxyhemoglobin by Beer-Lambert Law (Huppert et al., 2009). The short-distance channels were regressed from the long-distance channels to remove any interference originating from superficial layers. This was achieved by using a consecutive sequence of Gaussian basis functions ($stdev = 1\mu Mol$; $step = 1s$) via ordinary least squares to regress scalp and dura activation data collected from the short separation fibers, to create the hemodynamic response function (HRF) (Huppert et al., 2009). Then, the time series data for each of the 28 channels were group-averaged into eight distinct regions of interest as follows, according to the anatomical structures: left PFC (source 1, detectors 1 and 2), medial PFC (source 2, detectors 2 and 3), right PFC (source 3, detectors 3 and 4), left lateral M1 (source 4, detectors 5 to 8 and 17), LMM1 (source 5, detectors 8 to 10), right medial M1 (source 6, detectors 9 to 12), right lateral M1 (source 7, detectors 11 to 14 and 18), and finally, SMA (source 8, detectors 9, 15, 16 and 19).

The time range used from the fNIRS time series to calculate the brain activation level was determined by Linear Discriminate Analysis (LDA). The LDA was used to classify three groups based on the averaged oxy-hemoglobin (HbO) value within time ranges, with an exhaustive grid search on the starting time point and ending time point, with an increment of 5s. Then we selected the time range of 10s – 40s, which yielded the largest LDA accuracy, as shown in Fig. S8.

2 Supplementary discussion

Existing literature supports our observations. M1 tDCS was observed to lower the hand path errors in an arm reaching task (Osu et al., 2003). In Ehsani et al.'s study (Ehsani et al., 2016), a significantly lowered error was seen in motor sequence learning by M1 tDCS. It is worth mentioning that a study (Ciechanski et al., 2018) also showed that tDCS was able to enhance the scores of the FLS pattern cutting task. However, their protocol only involved one-day training with eight repetitions of the pattern cutting task whereas, in our protocol, each participant practiced up to 10 trials per day from training day 2 to day 12, resulting in more than 100 repetitions. Therefore, our protocol involves various learning stages including acquisition, consolidation, and retention. Other previous studies have shown that learners could not reach proficiency in one day for laparoscopic skills (Louridas et

al., 2017). In our earlier analysis, even with the 12-day training on laparoscopic skills, some learners could not reach proficiency (Nemani et al., 2018; Gao et al., 2020). Here, we are the first to investigate the effect of tES during this 12-day training procedure. We did not see a significant difference in one training day, as in (Ciechanski et al., 2018), comparing tDCS to the Sham group (the results are found in Fig. S9, which could be compared to Ciechanski et al.'s results (Ciechanski et al., 2018)). The experimental settings are different in evaluating metrics (they assessed FLS score post-training, but we accessed the performance during the training) and tES settings (delivery timing: during the task in Ciechanski et al.'s study (Ciechanski et al., 2018); here before the task).

3 Supplementary Figures and Tables

3.1 Supplementary Figures

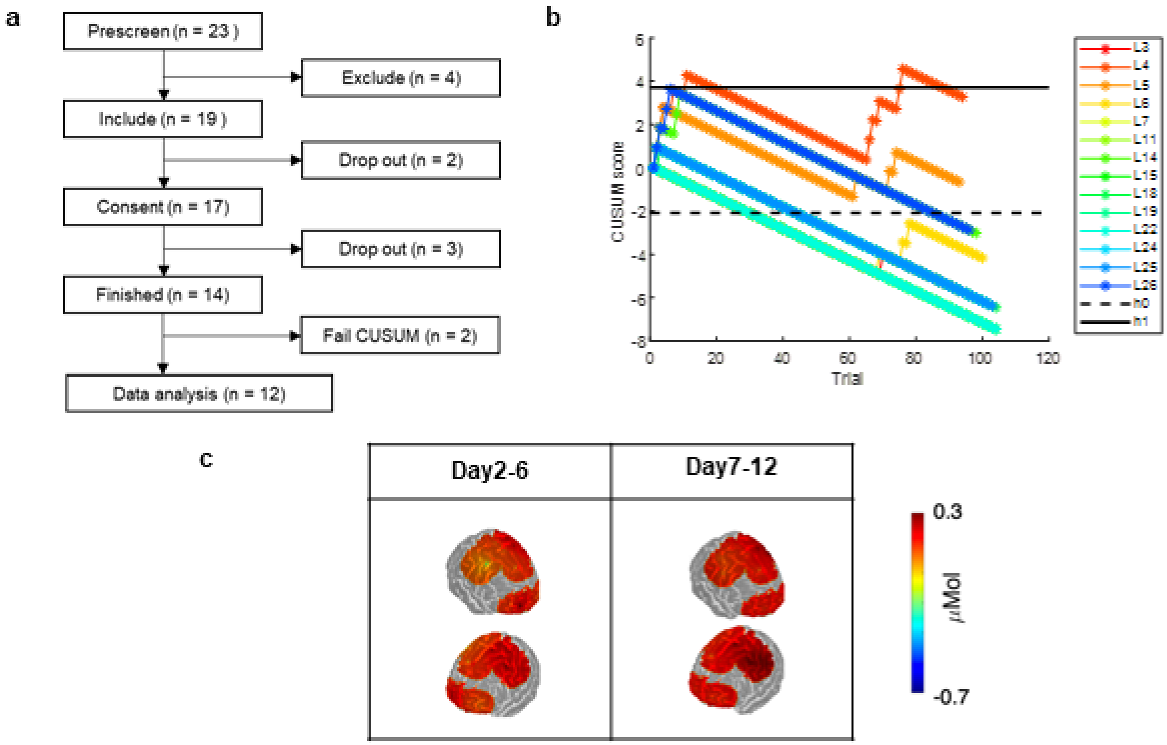

Supplementary Figure 1. Participant recruitment. (a) Participant recruitment procedure. (b) CUSUM scores for each trained subject with respect to trials. The h0 threshold indicates that the probability of any given trained subject is mislabeled as a "Skilled trainee" is less than 0.05 and is subsequently labeled as an "Skilled trainee" subject. Results indicate that 4 trained subjects, 'L2', 'L4', 'L5' and 'L21', are labeled as "Unskilled trainees." The remaining trained that cross the h0 line are labeled "Skilled trainees." (c) The brain activation map for the unskilled subjects whose data have been excluded from data analysis.

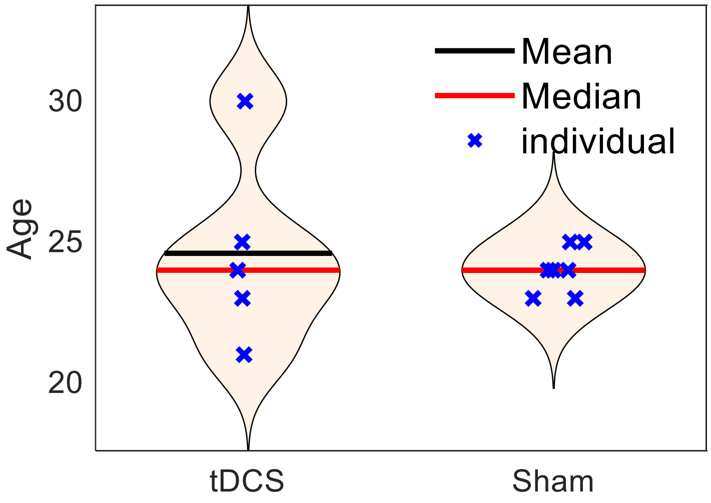

Supplementary Figure 2. Age distribution of participants for each group.

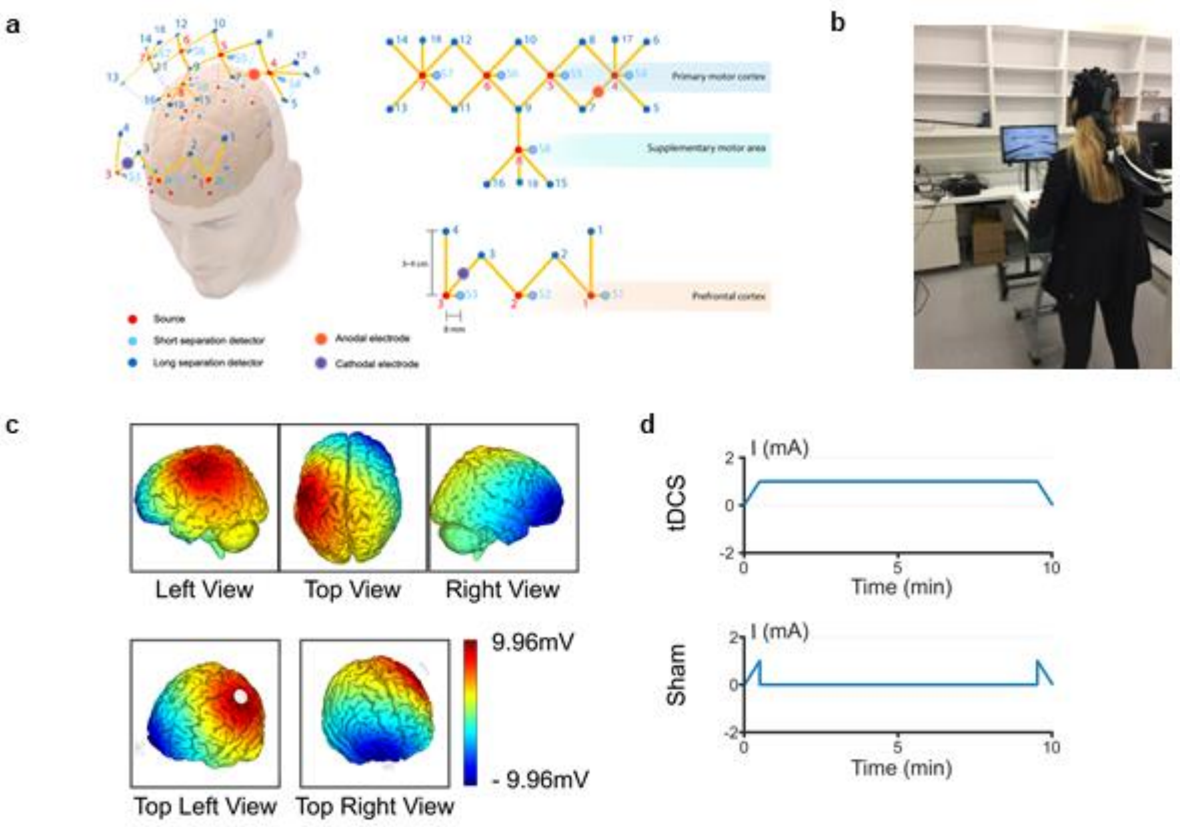

Supplementary Figure 3. fNIRS and tES setups. (a) Optode positions for coverage over the PFC, M1, and SMA. Small red dots indicate the locations of infrared sources; small blue dots indicate long separation detectors; small light blue dots indicate short separation detectors; large red dots indicate anodal electrode; large purple dot indicates cathodal electrode. The PFC has three sources (1 to 3), three short separation detectors (S1 to S3), and four long separation detectors (1 to 4). The M1 has 4 sources (4 to 7), 4 short separation detectors (S4 to S7), and 12 long detectors (5 to 14, 17, and 18). The SMA has one source (8), one short separation detector (S8), and 4 long separation detectors (9, 15, 16, and 19). (b) A photo of a subject performing the task with fNIRS and tES. (c) The electrical distribution field. (d) The electrical wave forms for tDCS and Sham.

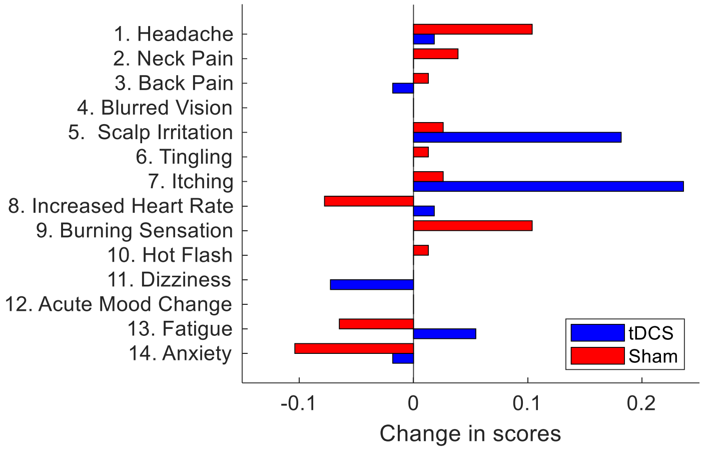

Supplementary Figure 4. The safety questionnaire data. The change in scores represents the score rated after the neuromodulation minus the score before. The scale is from 1 to 10, with 1 as the most absent whereas 10 as the most severe sensation.

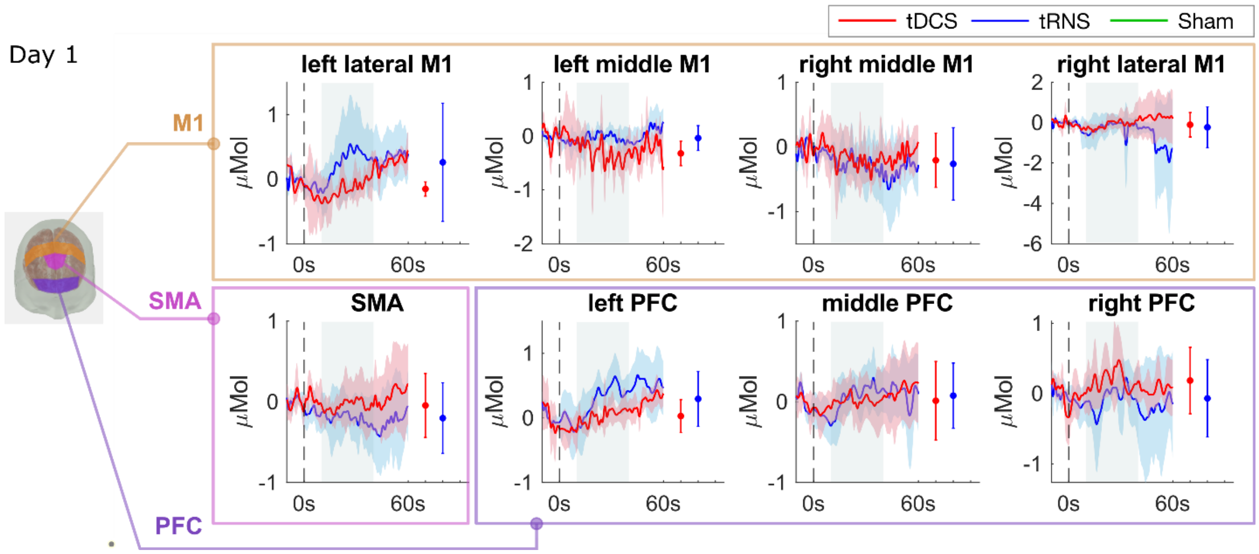

Supplementary Figure 5. Brain functional activation for training day 1. Grouped average time-series HRFs with respect to cortical regions on training day 1. The solid lines are mean values, and the shaded areas are 95% confidence interval. The stimulus onset begins at zero seconds (dashed black line) indicating that the trial has started. Negative time indicates the baseline measurement used for calibration before each trial. The grey painted box (10-40s) is the time range selected to calculated the mean HbO values. The mean and 95% CI of 10-25s HRFs are plotted next to the time-series HRFs in error bar form.

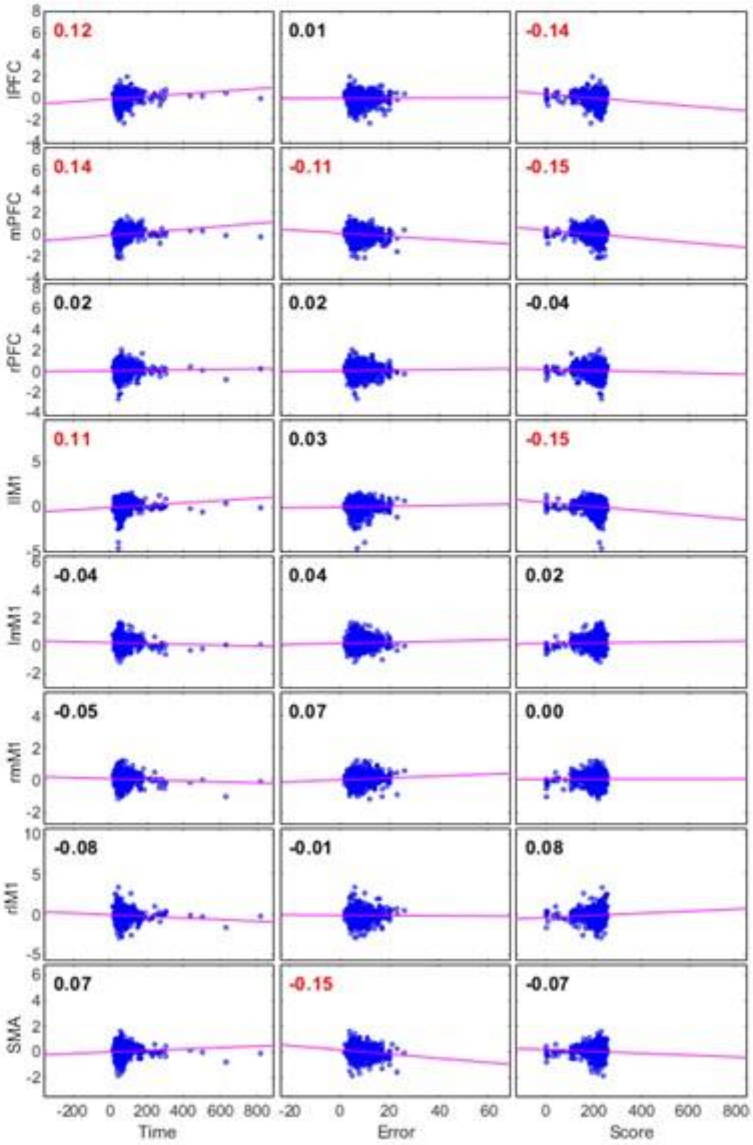

Supplementary Figure 6. Correlation analysis between the performance (time, error, score) and the brain activation in brain areas. The Pearson's correlation coefficients are labeled on each subplot.The significant r values are colored red. Alpha value was adjusted to 0.05/24 by Bonferroni correction.

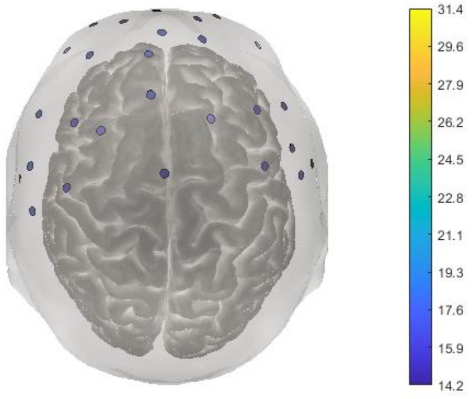

Supplementary Figure 7. The averaged optode position for fNIRS. The color represents the spatial standard deviation.

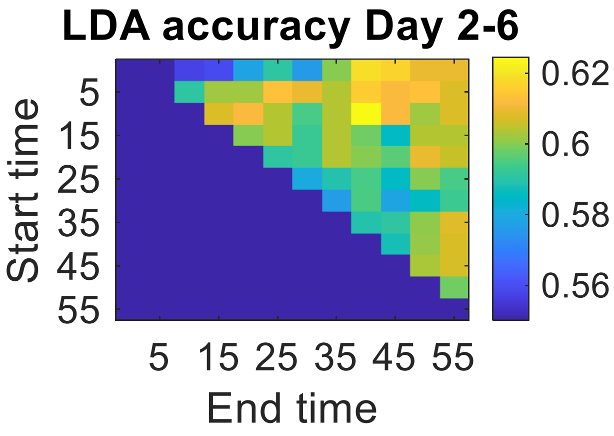

Supplementary Figure 8. Select the time range along the fNIRS time series data. (a) An example of the time series mean HbO value from the three groups. HbO rises at the beginning of the task performance and drops back. The time range (orange box) is when the fNIRS time series values diverge. (b) LDA model was adopted to classify the three groups based on the different time range of average HbO values, and the accuracy value was color-coded. Time range from 10s to 40s yields the highest accuracy value.

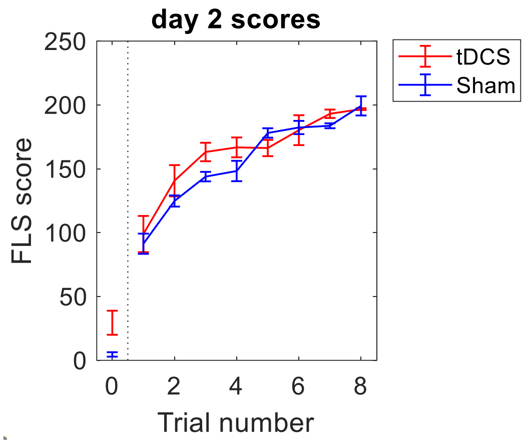

Supplementary Figure 9. The FLS score for each trial on day 2. Trial 0 is the baseline performance on day 1. The bar represent the standard deviation. The three groups are not significantly different in the FLS scores for any of the trials on day 2.

3.2 Supplementary Tables

Table S1. Comparison between tDCS and Sham groups on each day

Training day	Time	Error	Score	Std of time	Std of error	Std of score
Day1	p = 0.202	p = 0.609	p = 0.232	p = 1.000	p = 1.000	p = 1.000
Day2	p = 0.349	p = 0.879	p = 0.295	p = 0.639	p = 0.343	p = 0.755
Day3	p = 0.887	p = 0.243	p = 0.176	p = 0.755	p = 1.000	p = 0.755
Day4	p = 0.582	p = 0.027	p = 0.060	p = 0.343	p = 0.639	p = 0.343
Day5	p = 0.150	p = 0.795	p = 0.040	p = 0.432	p = 0.268	p = 0.530
Day6	p = 0.071	p = 0.037	p = 0.002*	p = 0.268	p = 0.268	p = 0.073
Day7	p = 0.028	p < 0.001*	p = 0.913	p = 1.000	p = 0.030	p = 0.432
Day8	p = 0.402	p < 0.001*	p = 0.043	p = 0.343	p = 0.343	p = 0.755
Day9	p = 0.310	p < 0.001*	p = 0.017	p = 0.755	p = 0.018	p = 0.202
Day10	p = 0.012	p = 0.002*	p = 0.898	p = 1.000	p = 0.876	p = 0.432
Day11	p = 0.111	p < 0.001*	p = 0.762	p = 0.432	p = 0.202	p = 0.268
Day12	p = 0.364	p < 0.001*	p = 0.333	p = 0.343	p = 0.465	p = 0.202
Follow up	p = 0.402	p = 0.124	p = 0.180	NA	NA	NA

Note: The normality of data was checked by Kolmogorov-Smirnov test. The significance test was t-test when the data were normally distributed or Mann-Whiteney U-test if not. '*' indicates where p value is larger than alpha value, where alpha value was corrected as 0.05/6=0.008 by Bonferroni correction.

Table S2. Significance test on brain activation data

Data	lPFC	mPFC	rPFC	llM1	lmM1	rmM1	rlM1	SMA
Day1	0.167	0.860	0.396	0.503	0.170	0.579	0.312	0.087
Day2-6	0.568	0.475	0.014	0.463	0.758	0.129	0.015	0.924
Day7-12	0.055	0.261	0.001*	0.939	0.354	0.703	< 0.001*	<0.001*
FollowUp	0.037	0.012	0.323	0.158	0.500	0.043	0.553	0.305

Note: The normality of data was checked by Kolmogorov-Smirnov test. The significance test was t-test when the data were normally distributed or Mann-Whiteney U-test if not. '*' indicates where p value is larger than alpha value, where alpha value was corrected as 0.05/8=0.006 by Bonferroni correction.

Table S3. Repeated measurement ANOVA on performance data

Dependent Variable	Source	df	F	p-value
Performance time	Time	1.157*	27.945	<0.001
	Stimulation	1	0.071	0.796
	Time*Stimulation	1.157*	2.534	0.136
Performance error	Time	3.013*	0.944	0.432
	Stimulation	1	2.830	0.123
	Time*Stimulation	3.013*	1.199	0.327
Performance score	Time	3.307*	114.367	<0.001
	Stimulation	1	0.024	0.879
	Time*Stimulation	3.307*	2.842	0.048

Note: Levene's test was carried out and all the groups of data passed the test. Mauchly's test of Sphericity was also carried out but the data did not pass so we used Greenhouse-Geisser Epsilon correction to adjust the degrees of freedom and indicated by '*'.

Table S4. Optode 3D positions and spatial standard deviations (mm).

X	Y	Z	Std
166.20	149.38	226.29	19.34
124.95	142.87	233.52	20.61
88.14	146.30	222.71	22.14
197.95	87.80	136.62	27.76
142.53	52.90	144.40	21.63
95.85	59.16	147.15	19.92
57.54	90.24	136.09	16.43
121.62	74.82	194.71	26.85
184.65	133.26	209.87	18.36
146.48	124.20	229.02	19.73
101.50	120.19	223.21	22.67
68.42	128.23	199.66	21.33
197.82	102.53	165.50	30.12
212.57	115.73	130.15	14.20
176.51	73.24	159.03	22.25
174.53	60.59	119.92	19.48
120.55	58.23	166.48	24.10
119.89	47.70	119.78	21.36
72.78	79.97	157.21	20.66
74.34	68.89	123.14	18.08
57.12	103.06	162.35	18.85
47.94	115.00	136.13	14.85
161.35	94.26	204.39	27.70
97.86	91.07	202.43	25.16

196.47	81.65	109.64	24.85
59.32	87.55	113.62	24.74
127.34	90.16	209.48	31.36

4 References

Chellali, A., Ahn, W., Sankaranarayanan, G., Flinn, J. T., Schwaitzberg, S. D., Jones, D. B., et al. (2015). Preliminary evaluation of the pattern cutting and the ligating loop virtual laparoscopic trainers. *Surg. Endosc.* 29, 815–821. doi:10.1007/s00464-014-3764-7.

Ciechanski, P., Cheng, A., Damji, O., Lopushinsky, S., Hecker, K., Jadavji, Z., et al. (2018). Effects of transcranial direct-current stimulation on laparoscopic surgical skill acquisition. *BJS Open* 2, 70–78. doi:10.1002/bjs5.43.

Ehsani, F., Bakhtiary, A. H., Jaberzadeh, S., Talimkhani, A., and Hajihasani, A. (2016). Differential effects of primary motor cortex and cerebellar transcranial direct current stimulation on motor learning in healthy individuals: A randomized double-blind sham-controlled study. *Neurosci. Res.* 112, 10–19. doi:10.1016/j.neures.2016.06.003.

Fraser, S. A., Klassen, D. R., Feldman, L. S., Ghitulescu, G. A., Stanbridge, D., and Fried, G. M. (2003). Evaluating laparoscopic skills, setting the pass/fail score for the MISTELS system. *Surg. Endosc. Other Interv. Tech.* 17, 964–967. doi:10.1007/s00464-002-8828-4.

Gao, Y., Kruger, U., Intes, X., Schwaitzberg, S., and De, S. (2020). A machine learning approach to predict surgical learning curves. *Surgery* 167, 321–327.

Huppert, T. J., Diamond, S. G., Franceschini, M. A., and Boas, D. A. (2009). HomER: a review of time-series analysis methods for near-infrared spectroscopy of the brain. *Appl. Opt.* 48, D280–D298.

Kestin, I. G. (1995). A statistical approach to measuring the competence of anaesthetic trainees at practical procedures. *Br. J. Anaesth.* 75, 805–809. doi:10.1093/bja/75.6.805.

Linsk, A. M., Monden, K. R., Sankaranarayanan, G., Ahn, W., Jones, D. B., De, S., et al. (2018). Validation of the VBLaST pattern cutting task: A learning curve study. *Surg. Endosc. Other Interv. Tech.* 32, 1990–2002. doi:10.1007/s00464-017-5895-0.

Louridas, M., Szasz, P., Fecso, A. B., Zywiel, M. G., Lak, P., Bener, A. B., et al. (2017). Practice does not always make perfect: Need for selection curricula in modern surgical training. *Surg. Endosc. Other Interv. Tech.* 31, 3718–3727. doi:10.1007/s00464-017-5572-3.

Nemani, A., Yücel, M. A., Kruger, U., Gee, D. W., Cooper, C., Schwaitzberg, S. D., et al. (2018). Assessing bimanual motor skills with optical neuroimaging. *Sci. Adv.* 4, eaat3807. doi:10.1126/sciadv.aat3807.

Osu, R., Burdet, E., Franklin, D. W., Milner, T., and Kawato, M. (2003). Different mechanisms involved in adaptation to stable and unstable dynamics. *J Neurophysiol* 90, 3255–3269.

Thair, H., Holloway, A. L., Newport, R., and Smith, A. D. (2017). Transcranial direct current stimulation (tDCS): A beginner's guide for design and implementation. *Front. Neurosci.* 11, 641. doi:10.3389/fnins.2017.00641.
